# When a smartphone‐free childhood is not a choice: Recognising smartphones as essential medical devices

**DOI:** 10.1016/j.clinme.2026.100591

**Published:** 2026-04-24

**Authors:** Alice M. Gregory, Partha Kar

**Affiliations:** a Department of Psychology, Royal Holloway, University of London, Egham, UK; b Portsmouth Hospitals NHS Trust, Portsmouth, UK

**Keywords:** Allergies, Asthma, Cardiology, Children, Digital health, Health equity, School policy, Smartphone, T1 diabetes, Young carers, Young people

## Abstract

Recently there have been initiatives to ban or reduce the use of smartphones in children and young people. While exemptions are typically made for those for whom smartphones constitute an essential medical device, these groups have not been sufficiently considered in discussions to date. These people include children living with type 1 diabetes and young carers, as well as some with heart conditions and epilepsy. Research urgently needs to examine the consequences of smartphone use and restriction in children in general, but this is particularly urgent when smartphones constitute an essential medical device. While restricting use and negative messaging surrounding campaigns could cause harm to these children, the negative impacts of devices (eg providing opportunities for bullying or distraction) may also be amplified in these populations. Four clinical and research recommendations on this topic are proposed.

Advances in digital technology have consequences for child development.^1^ Benefits include providing novel learning opportunities, additional ways to stay in touch with family and friends, ease of access to health information and digital mental health support.

However, there are also concerns – including around the childhood use of the smartphone, a device that provides a personal digital assistant – allowing complex computing with ease. There are questions about the addictive nature of these devices, the possibility that smartphone use displaces time for other activities such as socialising face to face, enjoying green and blue space, exercise and sleep. There are also serious risks associated with the content being viewed and novel challenges regarding bullying, grooming, negative self-comparison and other sources of harm.

Anxieties have also led to parental initiatives to ban smartphones in children and young people (eg smartphone-free childhood movements^2^). Political leaders have also supported the removal of these devices in schools.^3^ Movements of this type have been celebrated by some, although there is a paucity of research examining the consequences of different policies and research has produced mixed results.^4^

A piece of the puzzle that has received little research attention to date concerns those who use smartphones as essential medical devices. For some children, using a smartphone is not a choice, but is integral to their survival. Examples include children living with type 1 diabetes (T1D), where a smartphone can constitute an essential component of an automated insulin delivery system (or an ‘artificial pancreas’), which is often considered the gold standard in care. The function of the smartphone in the automated insulin delivery system depends on the specific device, but it can be used to monitor and control the insulin pump, for example. Smartphones also allow essential communication between caregivers and children in the management of a complex 24/7 condition for which there is often little support at school.^5^ Concerns have also been raised for young carers, with one charity reporting children needing to hide in toilets to have ʻsensitive and sometimes upsetting conversations with their family’.^6^ Children living with conditions including epilepsy, allergies, asthma and heart conditions can also use smartphones to support their condition.^7^, ^8^, ^9^

Although guidance to restrict smartphone use in young people typically includes exemptions for those who use these as essential medical devices, these can be confusing, inadequate, and risk being implemented poorly. For example, the Department for Education’s Guidance on Mobile Phones in School (2026) notes that, where reasonable adjustments are required, ʻSchools should develop practices which enable pupils to use their mobile phone for a specific purpose at specific times and locations, for example in a Head of Year’s Office’.^3^ Such guidance risks causing harm. For example, without detailed understanding of medical conditions, such as T1D, a school following this guidance might expect a child to check their blood glucose at specific times of day only (which would be inappropriate given that blood glucose is continuously fluctuating) and at a specific location (which could single them out, and be against medical recommendations during an episode of hypoglycaemia for example, where advice is not to move, but to ‘treat in the seat’).

Relevant to populations using smartphones as medical devices, we flag two concerns. First, there are risks associated with restricted use of an essential medical device (eg school bans; restricted use in specific areas). Second, there are concerns about some of the narrative surrounding these discussions (with condemnation of smartphone use in young people by respected adults).

Possible unexplored consequences of smartphone-free initiatives for children who use these devices and their families include:

1) physical harm (by the prevention or reluctance to use devices in a way that is useful or essential for the associated medical condition);
2) perceived stigma associated with smartphone use (and, by proxy, the medical conditions with which use is associated);
3) shame associated with smartphone use; mental health difficulties; divisions in society (with those who support bans and those who use devices for medical purposes); and
4) health disparities.

We make four research and clinical recommendations in this area (see also^5^):

First, research into the advantages and disadvantages of smartphone use in children should specifically consider those for whom these are essential medical devices. This is particularly salient because some of the concerns flagged about smartphone use in children may be particularly noteworthy for those who live with conditions requiring their use. As an example, concerns about smartphones causing distraction from learning in class may be particularly noteworthy in children living with T1D, who have been shown to have lower sustained attention compared to those without.^10^ Worries that smartphones can lead to greater opportunities for bullying around the clock may be particularly noteworthy in children living with chronic conditions, who report more discrimination than others.^11^

Second, research assessing consequences of restrictions and removal of smartphones in children should particularly consider impact on children who use these as essential medical devices, as well as their social networks, given the particular significance of these changes for these populations and the potential for negative consequences.

Third, there should be greater advocacy and greater awareness at every level concerning children who use smartphones as essential medical devices so that families are not left to navigate restrictions and bans of these devices alone. Recommendations include both school-level exemptions and implementation guidance. Support from politicians, schools, medics and the wider community is key.

Fourth, technology and medical development need to stay abreast of these societal changes and discussions, and there should be novel research to fully understand the implications of any future innovations. Indeed, research should examine whether the use of smartphones as medical devices impacts family choice of intervention (and associated clinical outcomes) as well as perceived acceptability. Policy must be aligned with future innovation and advances.

Research and advocacy of this type is essential to put us in the best position to ensure that children, already living in challenging circumstances, do not get lost or disadvantaged in these societal discussions and changes happening right now.

## CRediT authorship contribution statement

**Alice M. Gregory:** Writing – original draft. **Partha Kar:** Writing – review & editing.

## Funding

There was no funding for this opinion piece.

## Declaration of competing interest

The authors declare the following financial interests/personal relationships which may be considered as potential competing interests: AMG is an adviser for a project initially sponsored by Johnson’s Baby. She was a consultant for Perrigo (2021+). She receives royalties for two books, *Nodding Off* (Bloomsbury Sigma, 2018) and *The Sleepy Pebble* (Flying Eye, 2019) and a sleep gift (The Gift of Sleep, Lawrence King Publishers, 2023). She was previously a CEO of Sleep Universal LTD (2022). She is a regular contributor to *BBC Focus* Magazine and has contributed to other outlets (such as *The Conversation*, *The Guardian*, *Balance* Magazine, Psyche Aeon). She occasionally receives sample products related to sleep (eg- blue light-blocking glasses) and has given a paid talk to a business (Investec). She has contributed a paid article to Neurodiem. She is sometimes invited to give presentations for which expenses are paid or for which she receives a small stipend. She is a mother of a child living with type 1 siabetes.

If there are other authors, they declare that they have no known competing financial interests or personal relationships that could have appeared to influence the work reported in this paper.
